# The diagnostic and predictive value of LncRNA PANDAR in gastric cancer and its regulation of gastric cancer progression via miR-637

**DOI:** 10.1186/s41065-025-00511-7

**Published:** 2025-07-26

**Authors:** Weikang Su, Zhiling Zhong, Huazhi Li, Lingjia Meng, Xinqiang Zhong

**Affiliations:** 1 Department of Gastroenterology, The First Affiliated Hospital of Jinan University, Guangzhou, 510632 China; 2 Department of Gastroenterology, The Second Hospital of Dalian Medical University, Dalian, 116000 China; 3 General Surgery Department, Beijing Anzhen Hospital, Capital Medical University, Beijing, 100029 China; 4 Department of Emergency Medicine, Putuo Hospital, Shanghai University of Traditional Chinese Medicine, No.164, Lanxi Road, Putuo District, Shanghai, 200333 China; 5 Department of Gastrointestinal Surgery, Huanggang Central Hospital Affiliated to Yangtze University, Dabie Mountain Regional Medical Center, No. 6, Qi’an Avenue, Huangzhou District, Huanggang, 438000, China

**Keywords:** lncRNA PANDAR, miR-637, Gastric cancer, Diagnostic biomarker, Tumor progression

## Abstract

**Background:**

Gastric cancer (GC) remains a global health challenge due to its high morbidity, late-stage diagnosis, and limited therapeutic options. long non-coding RNA PANDAR has been implicated in tumorigenesis across multiple cancers, yet its role in GC pathogenesis and diagnostic utility is still unclear. The purpose of this study is to elucidate the diagnostic value of serum PANDAR and its regulatory mechanisms in GC progression.

**Methods:**

lncRNA PANDAR levels were quantified in serum from 112 GC patients and 98 benign controls using RT-qPCR. The diagnostic value of lncRNA PANDAR was analyzed by ROC curve. Functional experiments (CCK-8, Transwell assays) and dual-luciferase reporter assays were conducted in HGC-27 and BGC-823 cells to explore the function of PANDAR in cell proliferation, migration, and invasion. The interaction between PANDAR and miR-637 was validated via bioinformatics prediction and co-inhibition assay.

**Results:**

Serum PANDAR was significantly upregulated in GC patients (*P* < 0.0001) and exhibited high diagnostic accuracy (AUC = 0.913). Elevated PANDAR associated with advanced TNM stage (*P* = 0.047), lymph node metastasis (*P* = 0.023), and digestive system history (*P* = 0.035). PANDAR knockdown suppressed GC cell proliferation, migration, and invasion, effects reversed by co-inhibition of miR-637.

**Conclusions:**

lncRNA PANDAR is a promising non-invasive diagnostic indicator of GC, and may serve as a biomarker for GC diagnosis and therapy. lncRNA PANDAR regulates the progression of GC through sponge miR-637.

## Background

Gastric cancer (GC) is a leading cause of upper gastrointestinal malignancy. According to the Cancer Statistics 2024 report, there are over one million new cases each year, with a five-year survival rate of only 30% [1]. Due to the non-specific nature of early clinical manifestations in gastric cancer, it is often confused with benign diseases such as chronic gastritis and peptic ulcer, resulting in about 70% patients being in the middle and late stage when they are diagnosed [2]. Currently, the definitive diagnosis of GC primarily relies on endoscopic biopsy, while its invasive nature poses risks of procedural complications, and lacks sensitivity in the diagnostic of early-stage lesions [3]. Therefore, the discovery of non-invasive biomarkers with high sensitivity and specificity holds significant promise for improving early detection rates, prognostic stratification, and therapeutic target development in gastric cancer [4]. The comprehensive review by the National Institutes of Health (NIH) outlines a paradigm shift in cancer treatment-from the emergence of surgical resection and radiotherapy to the development of targeted therapies and immunotherapies [5]. Notably, this evolution underscores the critical role of biomarkers in patient population stratification and tailoring interventions to molecular subtypes. Additionally, emerging therapies such as gene-targeted treatments and stem cell therapies are also recognized as pivotal strategies in cancer care, emphasizing the importance of identifying novel therapeutic targets and predictive biomarkers to guide clinical decision-making [6, 7].

Previously, numerous researchers have explored cancer biomarkers. The Liu laboratory has long been dedicated to pan-cancer research, aiming to uncover the clinical value of cancer biomarkers. Their team previously investigated Disulfidptosis-related genes through systematic characterization of over 9,000 samples across more tumor progression, thereby confirming their potential as candidate biomarkers for diagnosis, prognostic assessment, and targeted therapy [8]. Additionally, studies on TRPM7, based on multi-cancer lineage expression pattern analysis, have validated its reliability as a diagnostic biomarker [9]. Recently, the team further focused on voltage-gated sodium channels (VGSCs), demonstrating their dual diagnostic and therapeutic potential in breast cancer, colon cancer, prostate cancer, and lung cancer [10]. Collectively, these findings underscore that cancer biomarkers serve as a critical bridge connecting basic research and clinical applications, spanning early screening, precise molecular subtyping, treatment efficacy prediction, and prognostic evaluation, thus providing core support for advancing cancer diagnosis and treatment toward precision.

Long noncoding RNAs (lncRNAs) represent a heterogeneous class of nonprotein-coding biomolecules characterized by a transcript length exceeding 200 nucleotides [11]. lncRNAs exert their modulatory effects on disease progression through multilevel mechanisms including epigenetic regulation, and post-transcriptional modifications [12]. Many studies have shown that lncRNAs exhibit pervasive dysregulation in diverse cancers, serving as pivotal drivers of carcinogenesis [11]. lncRNA PANDAR, an antisense lncRNA transcribed from the CDKN1A locus, coordinates cellular responses to DNA damage through its regulatory activity, demonstrating aberrant upregulation across multiple solid tumors and showing strong correlation with malignant progression [13]. For example, the high expression of PANDAR was related to the decrease of overall survival (OS) of patients with colorectal cancer (HR = 3.43, 95% CI = 2.06–5.72) [14]. In addition, PANDAR was highly expressed in hepatocellular carcinoma tissues and cell lines, and its level was positively correlated with liver cirrhosis, hepatitis B surface antigen (HBsAg), alpha-fetoprotein (AFP), tumor nodule formation, vascular infiltration and TNM staging [15]. Notably, multiple clinical studies have corroborated that PANDAR exhibits significant clinical relevance in GC: In 2018, Gao S et al. conducted a meta-analysis involving over 6,000 GC patients and 51 lncRNAs from various articles. The results showed that the upregulation of PANDAR expression predicts poor prognosis of GC by meta-analysis (HR = 3.11, 95% CI = 2.72–3.55), hence PANDAR is a powerful candidate gene for predicting the prognosis of GC patients [16]. Moreover, Yang Z et al. measured plasma PANDAR levels of 109 GC patients and 106 healthy controls and conducted logistic regression analysis. The results indicated that PANDAR expression was significantly associated with clinicopathological parameters of GC, such as pathological differentiation and TNM stage (*P* < 0.05), which is suggested that it may be a potential marker of liquid biopsy [17]. Besides, in 2022, a study analyzed the expression levels of lncRNAs in the plasma of 51 breast cancer patients, the findings revealed a significant correlation between plasma PANDAR expression and family history in patients with metastatic breast cancer [18]. Based on previous studies, PANDAR expression levels demonstrate a strong correlation with GC progression, and we speculate that it may be a potential biomarker of GC. However, the precise molecular pathways through which PANDAR contributes to GC and its regulatory network with other non-coding RNA still need to be further analyzed.

microRNAs (miRNAs) as another key gene expression regulator, can participate in tumor progression by targeting oncogenes or tumor suppressor genes [19]. Among them, reduced expression level of miR-637 was consistently observed in GC tissues and cell lines, and its expression levels were closely correlated with tumor size, invasive depth, and patient survival rates [20]. Functional studies revealed that ATG7 and Beclin-1 expression levels are significantly reduced in GC cells following miR-637 overexpression, leading to autophagic inhibition [20]. Concurrently, miR-637 targeted the AKT/STAT3 signaling pathway to suppress cell proliferation and induce apoptosis [21]. Interestingly, emerging studies have demonstrated that PANDAR functions as a “molecular sponge” to adsorb miR-637, thereby relieving its inhibitory effects on downstream target genes [22]. For instance, in thyroid cancer, PANDAR exhibits a negative correlation with miR-637 expression. Overexpression of miR-637 suppresses thyroid cancer progression, an effect that can be reversed by PANDAR overexpression [22]. Whether this mechanism also exists in GC and how PANDAR/miR-637 axis affects the biological behavior of gastric cancer are still unclear.

Based on the above background, this research is designed to systematically investigate the diagnostic performance and predictive value of PANDAR in GC, while further elucidating the molecular mechanisms by which PANDAR regulates miR-637 to influence malignant phenotypes of gastric cancer cells. These findings highlight the potential of innovative non-invasive biomarkers in improving early gastric cancer diagnosis.

## Methods

### Study subjects

This study enrolled 112 patients with GC and 98 patients with benign gastric diseases at Putuo Hospital, Shanghai University of Traditional Chinese Medicine, and served as the study group and the control group respectively. All patients provided written informed consent prior to participation, and the study protocol was reviewed and approved by an independent ethics committee affiliated with the hospital. Collected basic information of all enrolled patients.

Inclusion and Exclusion Criteria:

1. Patients are diagnosed with GC by pathology.
2. No patients received preoperative chemotherapy or radiotherapy.
3. No other malignant lesions.
4. Exclude female patients during pregnancy and lactation.
5. Have complete medical and laboratory records.

### Serum sample collection

Collect 5 mL venous blood from patients in study group (*n* = 112) and control group (*n* = 98) respectively. After the blood sample was left standing at 4 ℃ for 30 min, it was centrifuged at 3000 rpm for 15 min. The isolated serum was aliquoted into RNase/DNase-free microtubes and cryopreserved at -80 ℃ for subsequent detection.

### Cell culture

The GC cell lines (HGC-27 and BGC-823) and a human gastric mucosal epithelial cell (GES-1) were purchased from ATCC (USA). Cells were cultured in the RPMI-1640 medium containing 10% fetal bovine serum and 1% penicillin/streptomycin, incubated at 37 ℃ with 5% CO_2_.

### Cell transfection

The PANDAR-specific siRNA and negative control siRNA were commercially synthesized by GenePharma (Shanghai) for this investigation. miR-637 mimic and inhibitor were purchased from GenePharma (Shanghai). Cell cultures were maintained in 24-well plates for 24 h before transfection, with confluence levels reaching 70–90% as confirmed by optical microscopy. The siRNA was mixed gentlywith 50 µL of Opti-MEM medium. Simultaneously, mixed 0.75 µL of Lipofectamine 3000 transfection reagent (Invitrogen, USA) and 25 µL of Opti-MEM medium gently. Incubated them for 5 min. Combined the two diluted solutions in a 1:1 ratio evenly and incubated at room temperature for 15 min. Transfered the mixed solution to each well of a 24-well plate and incubated at 37 °C. After 8 h, the liquid was changed to RPMI-1640 medium with 10% serum to continue the culture. miR-637 mimic/inhibitor and NC inhibitor were transfected into GC cells by Lipofectamine 3000 (Invitrogen, USA), similarly.

### Real-time quantitative PCR

Total RNA was isolated from serum using TRIzol reagent (Thermo Fisher Scientific, USA) following a standardized protocol. Synthesis of cDNA using PrimeScript™ RT reagent Kit (TaKaRa, Japan). The Mx3000P real-time PCR system (Thermo) was used to conduct real-time PCR, with 40 amplification cycles set at 94°C for 15 s, 60°C for 10 s, and 72°C for 20 s. The primer sequence is as follows: PANDAR, forward 5’-CTCCATCATGCCAAGTTCTGC-3’ and reverse 5’-GAAGGCAGGCAAGACTCGAA-3’; miR-637, forward 5’-GCTTTCGGGCTCTGCG-3’ and reverse 5’-GAACATGTCTGCGTATCTC-3’; U6, forward 5’-CTCGCTTCGGCAGCACAT-3’ and reverse 5’-TTTGCGTGTCATCCTTGCG-3’. Transcript quantification was performed using the 2^−ΔΔCT^ method.

### Cell proliferation assay

The CCK-8 assay (Beyotime, Shanghai) was employed to determine cell viability in this study. GC cells were seeded at 5 × 10³ cells/well in 96-well plates, exposed to 10 µL CCK-8 solution at 24, 48, and 72 h post-plating. Following 4-hour incubation at 37 ℃, absorbance at 450 nm was determined using the GloMax-Multi Detection System (Promega, USA).

### Cell metastasis assay

Cell suspensions were prepared in serum-free medium and subsequently loaded into the upper Transwell chambers for migration assays. The lower chamber contained complete culture medium with 10% FBS. The cells were cultured at a constant temperature of 37 ℃ for 48 h. Following incubation, cells localized in the bottom of the well chamber were immobilized using 4% paraformaldehyde for 10 min, followed by staining with 0.1% crystal violet solution. Cell counting was performed using an inverted optical microscope (Olympus, USA).

### Dual-luciferase reporter assay

Computational analysis predicted the potential binding sites between PANDAR and miR-637 using bioinformatics tools and subsequently cloned into the pmirGLO luciferase reporter vector (Promega, USA) to construct the wild-type PANDAR vector. Site-directed mutagenesis was performed on the binding site to generate the mutant-type PANDAR vector. Co-transfection of the constructed vectors with miR-637 mimic, miR-637 inhibitor, or negative controls were carried out using Lipofectamine 2000 (Invitrogen, USA). The Dual-Luciferase Reporter Assay System (Promega, USA) was employed to quantify firefly luciferase activity, normalized to Renilla luciferase activity as an internal control.

### Statistical analysis

The analytical dataset was processed through SPSS 26.0 and GraphPad Prism 9.0 for statistical evaluation, and the results were expressed as mean ± SD. According to the median expression level of PANDAR, GC patients were divided into high expression group and low expression group. Clinicopathological data from enrolled patients were evaluated by Chi-square test. The diagnostic value of PANDAR was analyzed by ROC curve. Logistic regression analysis to screen independent risk factors of GC. The independent samples t-test was selected to compare two experimental groups, whereas one-way ANOVA coupled with Tukey’s test was implemented to analyze multiple group variations, adhering to a significance threshold of *P* < 0.05.

## Results

### Baseline patient characteristics

This study collected the basic information of patients with GC (*n* = 112) and benign gastric lesions (*n* = 98), including age, gender, BMI, smoking history, drinking history, digestive system history, tumor size, differentiation degree, TNM staging, lymph node metastasis status and Lauren classification (Table 1). The study findings reveal a significantly higher prevalence of digestive system disease history among GC patients compared to those with benign gastric lesions (*P* = 0.019). Besides, there is no statistical difference in the general data of the two groups, which proves that the basic data of the two groups are matched and comparable. Among GC patients, the following characteristics demonstrated higher prevalence: tumor size < 5 cm (63.39%), middle-high differentiation (69.64%), TNM stage I classification (41.07%), lymph node metastasis (51.79%), and Lauren intestinal type (76.79%).

**Table 1:** The basic information of all subjects

Factors	Control	GC	*P* value
	(*n* = 98)	(*n* = 112)	
Age (years)	56.22 ± 12.95	55.42 ± 11.40	0.129
Gender (Male, %)	51 (52.04)	63 (56.25)	0.541
BMI (kg/m^2^)	21.01 ± 1.67	21.27 ± 1.85	0.392
Smoking History (yes, %)	21 (21.43)	32 (28.57)	0.235
Drinking History (yes, %)	27 (27.55)	39 (34.82)	0.258
History of digestive system (yes, %)	26 (26.53)	47 (41.96)	0.019
Tumor Size (n, %)			-
< 5 cm	-	71 (63.39)	
≥ 5 cm	-	41 (36.61)	
Differentiation (n, %)			-
Low	-	34 (30.36)	
Middle-High	-	78 (69.64)	
TNM stage (n, %)			-
I II	- -	46 (41.07) 39 (34.82)	
III IV	- -	16 (14.29) 11 (9.82)	
LNM (n, %)			-
Negative	-	54 (48.21)	
Positive	-	58 (51.79)	
Lauren Classification (n, %)			-
Intestinal Type	-	86 (76.79)	
Diffuse Type	-	26 (23.21)	

Notes: BMI: body mass index; TNM: tumor node metastasis; LNM: lymph node metastasis; *P* < 0.05 means a significant difference

### Expression of PANDAR in serum and its diagnostic significance

**Fig. 1:**
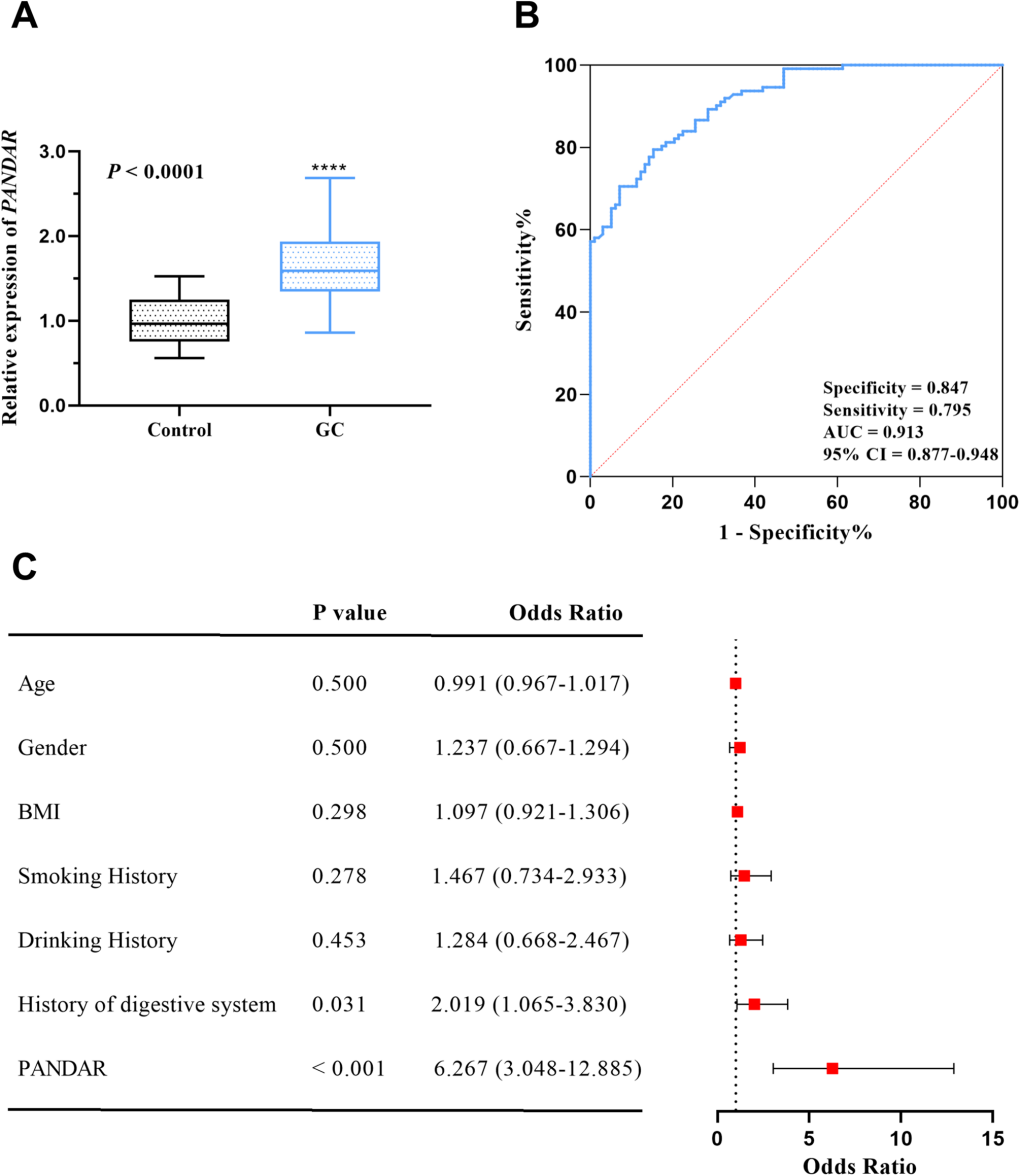
Expression and significance of PANDAR in GC. (**A**) Serum PANDAR levels demonstrated a marked elevation in GC patients compared to control group (*P* < 0.0001). (**B**) ROC curve showed that PANDAR has diagnostic value. (**C**) Logistic analysis showed that the up-regulation of PANDAR expression was a risk factor for GC

The expression level of PANDAR in serum of patients with GC was significantly higher than that of patients in control group (*P* < 0.0001, Fig. 1A). ROC curve analysis demonstrated the diagnostic efficacy of PANDAR, achieving an area under the curve of 0.913 (95% CI = 0.877–0.948, Fig. 1B). Logistic regression analysis identified history of digestive system (*P* = 0.031, OR = 2.019, 95% CI = 1.065–3.830) and PANDAR (*P* < 0.001, OR = 6.267, 95% CI = 3.048–12.885, Fig. 1C) as independent risk factors for GC progression in patients with benign gastric polyps. For the logistic regression model predicting metastasis, the Hosmer-Lemeshow goodness-of-fit test yielded a chi-squared statistic of 10.463 (df = 8, *P* = 0.234), indicating adequate calibration. The Cox-Snell pseudo-R^2^ was 0.179, and the Nagelkerke pseudo- R^2^ was 0.239, suggesting that PANDAR expression explained approximately 17.9-23.9% of the variance in metastasis risk.

### Relationship between PANDAR and clinical characteristics of GC patients

Based on the median serum PANDAR expression, GC patients were categorized into high-expression and low-expression groups. The expression of PANDAR was significantly correlated with the history of digestive system (*P* = 0.035), TNM stage (*P* = 0.047) and lymph node metastasis (*P* = 0.023) of patients (Table 2).

**Table 2:** Association of PANDAR expression and patients’ clinical features

	Cases (*n* = 112)	PANDAR expression		*P* value
		Low	High	
Age (years)				0.563
< 60	67	32	35	
≥ 60	45	24	21	
Gender				0.341
Male	63	34	29	
Female	49	22	27	
Smoking History				0.676
No	80	41	39	
Yes	32	15	17	
Drinking History				0.321
No	73	39	34	
Yes	39	17	22	
History of digestive system				0.035
No	65	38	27	
Yes	47	18	29	
Tumor Size				0.078
< 5 cm	71	40	31	
≥ 5 cm	41	16	25	
Differentiation				0.100
Low	34	13	21	
Middle-High	78	43	35	
TNM stage				0.047
I-II	85	47	38	
III-IV	27	9	18	
LNM				0.023
Negative	54	33	21	
Positive	58	23	35	
Lauren Classification				0.179
Intestinal Type	86	46	40	
Diffuse Type	26	10	16	

### Effect of PANDAR knockdown on the proliferation, migration and invasion of GC cells

**Fig. 2:**
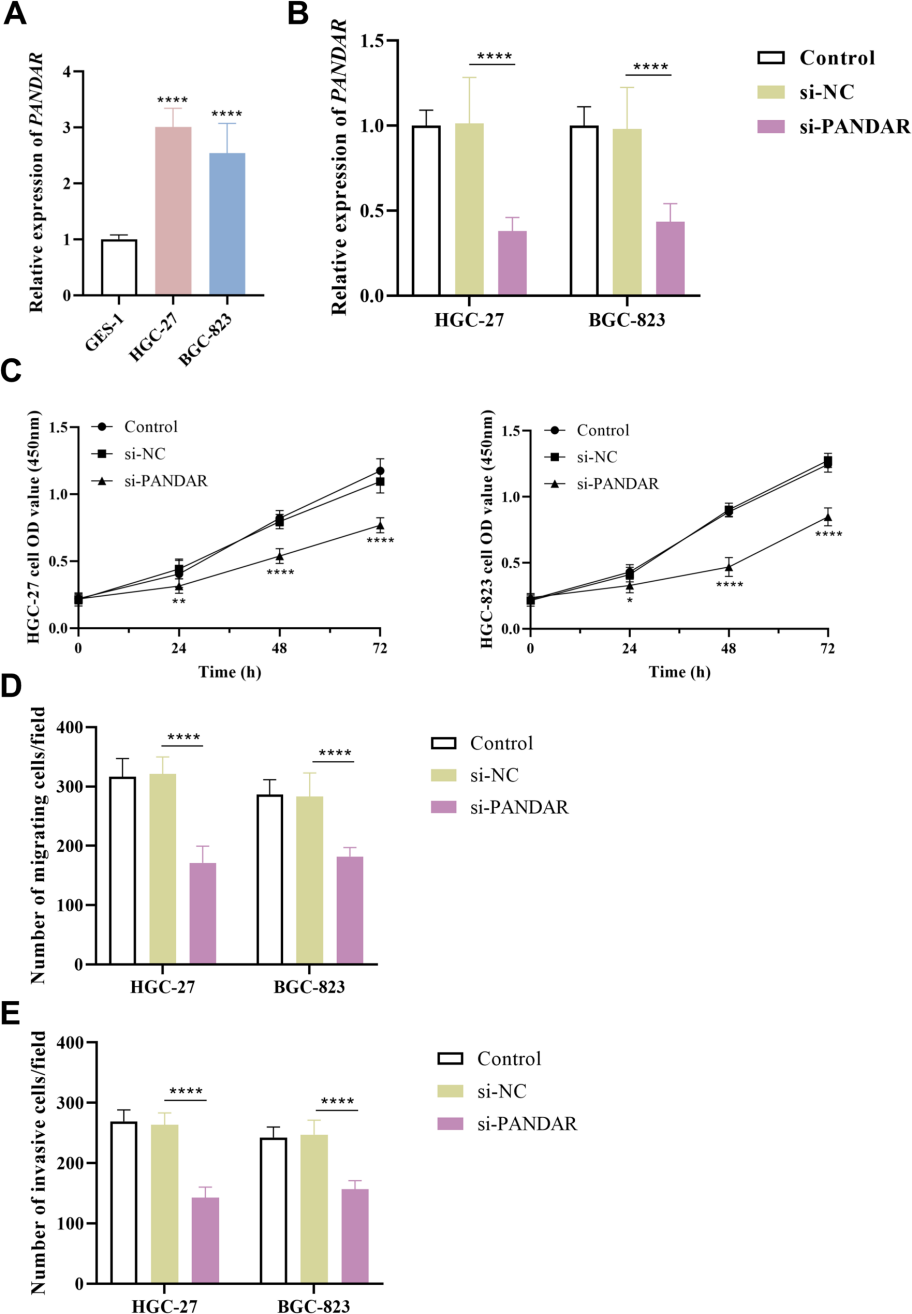
The function of PANDAR in GC cellular processes. (**A**) PANDAR expression was significantly higher in GC cells. (**B**) Transfection of si-PANDAR into HGC-27 and HGC-823 cells could inhibit the expression of PANDAR. (**C**) si-PANDAR inhibited the proliferation of both HGC-27 and HGC-823 cells. **D-E.** si-PANDAR markedly inhibited the migration (**D**) and invasion (**E**) of HGC-27 and HGC-823 cells. *P < 0.05, **P < 0.01, ****P < 0.0001

PANDAR exhibited markedly elevated expression levels in GC cell lines (HGC-27 and HGC-823) compared to normal human gastric mucosal epithelial cells (GES-1, *P* < 0.0001, Fig. 2A). After transfecting si-PANDAR into GC cells, RT-qPCR was used to detect the interference efficiency, revealing that si-PANDAR group showed significantly lower levels than si-NC group (*P* < 0.0001, Fig. 2B). Moreover, si-PANDAR significantly inhibited the activity of HGC-27 and HGC-823 cells (*P* < 0.0001, Fig. 2C), and similarly suppressed their migration (*P* < 0.0001, Fig. 2D) and invasion (*P* < 0.0001, Fig. 2E).

### The regulatory effects of PANDAR on miR-637

**Fig. 3:**
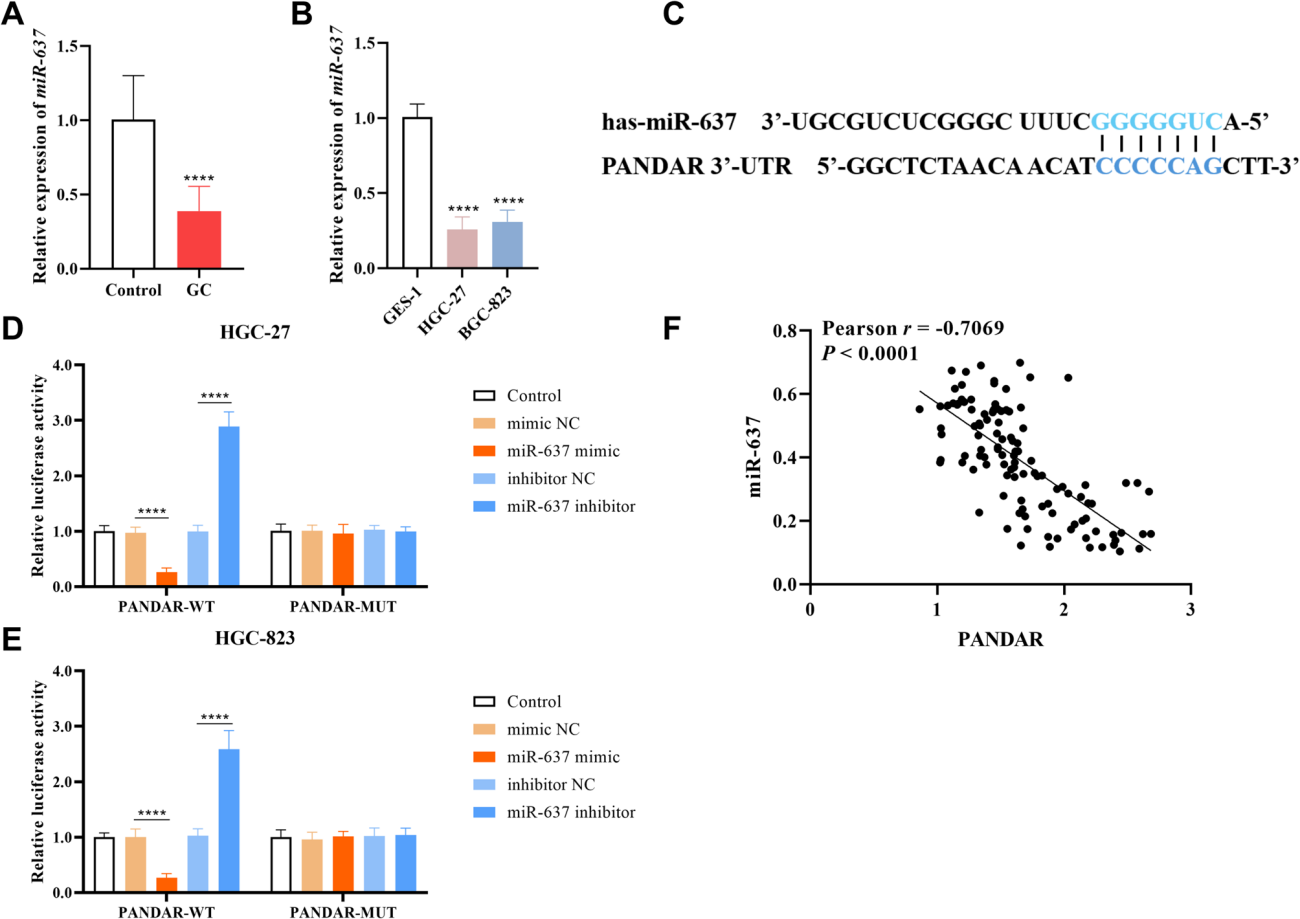
Expression of miR-637 and its targeted binding to PANDAR. **A-B.** The expression of miR-637 was significantly up-regulated in serum of patients with GC (**A**) and in GC cells (**B**). (**C.**) The predicted binding sites between PANDAR and miR-637. **D-E.** The dual-luciferase reporter assay validated the direct binding between PANDAR and miR-637 in HGC-27 (**D**) and HGC-823 cells (**E**). (**F**) miR-637 showed a negative correlation with PANDAR (*P* < 0.0001). ****P < 0.0001

Serum miR-637 levels demonstrated a marked downregulation in GC patients compared to healthy controls (*P* < 0.0001, Fig. 3A), and similarly, its expression level was also significantly reduced in HGC-27 and HGC-823 cells (*P* < 0.0001, Fig. 3B).

The interaction interfaces between PANDAR and miR-637 were predicted using the ENCORI database (Fig. 3C). In HGC-27 cell, miR-637 mimic transfection reduced PANDAR-WT luciferase activity compared to mimic NC (*P* < 0.0001), while miR-637 inhibitor increased activity versus inhibitor NC (*P* < 0.0001). PANDAR-MUT constructs abolished these effects (*P* > 0.05, Fig. 3D). Similar results were also found in HGC-823 cells: in PANDAR-WT, the miR-637 mimic induced significant suppression of luciferase activity compared to the mimic NC group (*P* < 0.0001), but the transfection of miR-637 inhibitor prominently increased the luciferase activity compared with inhibitor NC (*P* < 0.0001, Fig. 3E). To further validate the regulatory effect of PANDA on miR-637 expression, a two-tailed Pearson correlation analysis was performed to systematically evaluate the expression correlation between the two molecules in patients with GC (*n* = 112). The results demonstrated a significant negative correlation between serum PANDAR levels and miR-637 levels (*r* = -0.7069, *P* < 0.0001, Fig. 3F). Therefore, these results could prove that PANDAR can directly target miR-637 and inhibit its expression.

### Impact of co-inhibiting PANDAR and miR-637 on GC cells

**Fig. 4:**
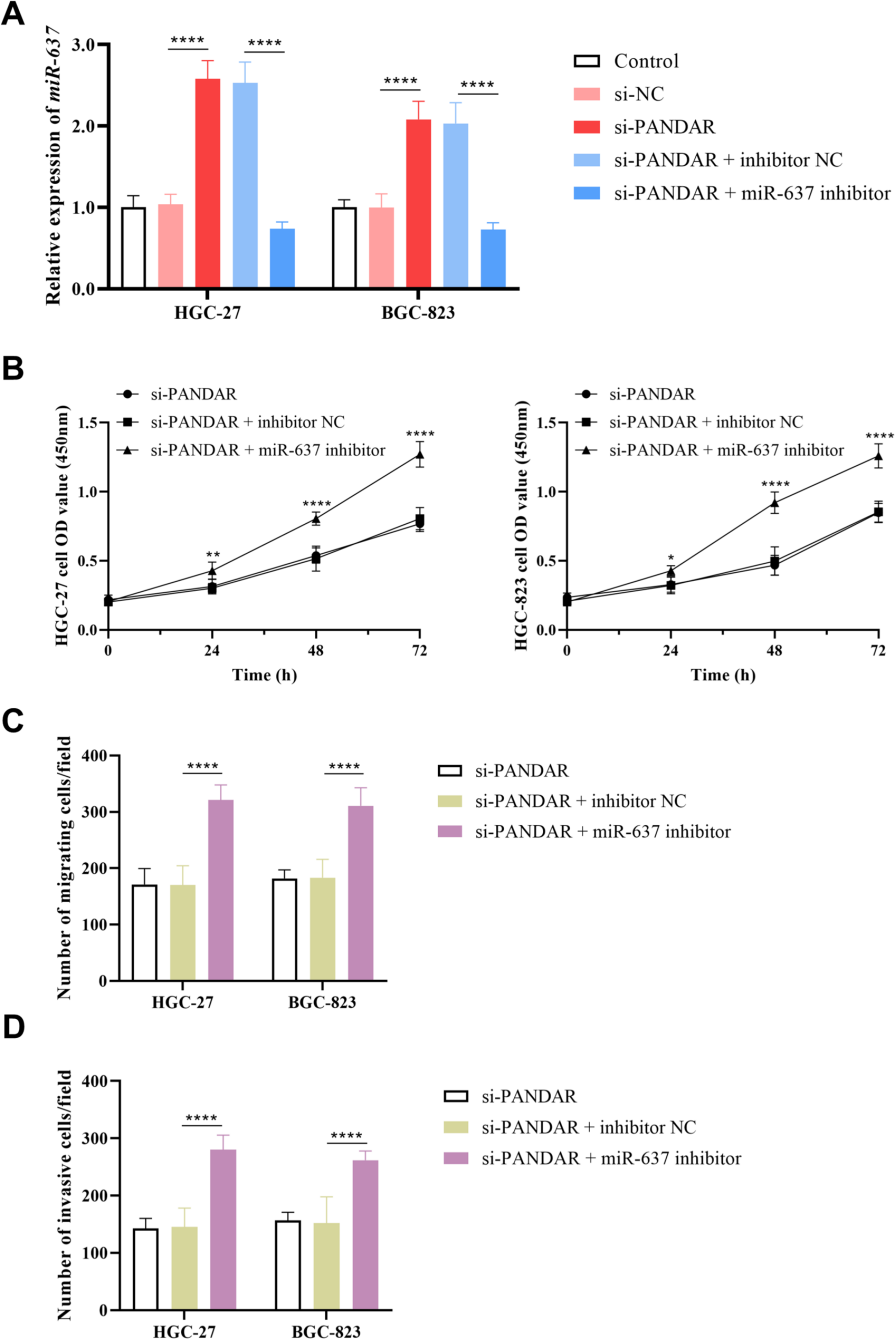
miR-637 mediated the effect of PANDAR on GC cellular processes. (**A**) si-PANDAR induced overexpression of miR-637, while miR-637 inhibitor reversed this effect. **B-D**: The inhibitory effects of PANDAR knockdown on the proliferation (**B**), migration (**C**), and invasion (**D**) of HGC-27 and HGC-823 cells were alleviated by miR-637 inhibitor. *P < 0.05, **P < 0.01, ****P < 0.0001

The knockdown of PANDAR led to overexpression of miR-637 in GC cells (*P* < 0.0001), while co-inhibition of PANDAR and miR-637 markedly reduces miR-637 expression in HGC-27 and HGC-823 cells (*P* < 0.0001, Fig. 4A). siRNA-mediated PANDAR silencing was combined with miR-637 inhibitor treatment to evaluate their synergistic impact on gastric cancer cell proliferation miR-637 could reverse the inhibitory effect of PANDAR on GC cells proliferation (*P* < 0.0001, Fig. 4B), suggesting its potential involvement in the regulatory mechanism of PANDAR.

Cell metastasis assay results demonstrated that transfection with miR-637 inhibitor caused a statistically significant increase in cell numbers in HGC-27 and HGC-823 cells. miR-637 functions as a suppressor of GC cells migration (*P* < 0.0001, Fig. 4C) and invasion (*P* < 0.0001, Fig. 4D) under physiological conditions, whereas its activity inhibition led to markedly enhanced metastatic potential.

## Discussion

Despite significant advancements in diagnostic and therapeutic technologies for GC in recent years, its mortality rate remains persistently high globally, particularly in high-incidence regions such as East Asia [23]. The core of this paradox lies in the dual challenges of low early diagnosis rates and limitations in late-stage treatment efficacy. As early-stage GC typically lacks specific clinical manifestations, most cases are diagnosed at advanced stages [24]. In current clinical practice, GC screening predominantly relies on invasive diagnostic procedures, which are typically initiated only after symptomatic presentation, thereby exacerbating diagnostic delays in disease detection [25]. Therefore, the discovery of emerging biomarkers and the exploration of diagnostic and therapeutic targets are particularly crucial. This study investigated the expression level of PANDAR in serum and its diagnostic and predictive value for GC. Compared with previous studies, it has the advantages of non-invasive and high efficiency. As a member of the non-coding RNA family, LncRNA PANDAR exhibits high sensitivity to dynamic alterations in the tumor microenvironment, functioning as a molecular diagnostic biomarker that provides a potential diagnostic target for early disease detection. In addition, logistic regression analysis identified PANDAR as a risk factor for GC, so we concluded that PANDAR has the potential as a diagnostic and predictive marker for GC.

This study showed that the overexpression of PANDAR is connected with clinical features of patients with GC, including the history of digestive system, TNM stage and lymph node metastasis. Previous studies have demonstrated that GC development arises from multifactorial synergy, with the TNM stage serving as an important indicator of GC progression [26]. Furthermore, digestive system history, such as helicobacter pylori infection, also could elevate the risk of carcinogenesis [27]. Therefore, the high expression of PANDAR demonstrates a strong correlation with GC biological characteristics, providing compelling evidence for its critical role in GC pathogenesis and progression.

The abnormal proliferation of GC cells directly drives the tumor volume expansion and local infiltration, while the migration and invasion ability promote the formation of metastatic lesions, which together constitute the core driving force of malignant progress [28]. It has been previously reported that PANDAR modulates diverse molecular mechanisms underlying carcinogenesis. For example, Li Y et al. found that inhibition of PANDAR can reduce the proliferation, migration and invasion of breast cancer cells [29]. These findings are consistent with our previous experimental results: the silencing of PANDAR suppressed GC cells proliferation, migration and invasion, suppressing tumor progression, suggesting that PANDAR can promote GC carcinoma cell proliferation. Therefore, PANDAR acts as a cancer-promoting factor in GC cells, driving tumorigenesis and metastatic progression.

LncRNAs function as miRNA sponges by binding to miRNAs through their structural domains, thereby inhibiting miRNA-mediated repression of target genes and dynamically modulating downstream signaling pathways. Qing Y et al. indicated that PANDAR modulates thyroid carcinoma proliferation and metastasis via miR-637 mediated regulation [22]. Based on previous studies, the regulatory mechanisms of the PANDAR/miR-637 axis in gastric carcinogenesis were investigated in this study. It is reported that miR-637, as a tumor suppressor gene of non-small cell lung cancer, inhibits the proliferation, migration and invasion of cancer cells [30]. miR-637 has also been proved to be involved in the development of gastric cancer and liver cancer [20, 31]. This study demonstrated that miR-637 reverses the inhibitory effects of PANDAR silencing on gastric cancer cell proliferation and metastasis, indicated that miR-637 is involved in regulating PANDAR-mediated processes in GC cells. Hence, we revealed that miR-637 as a key node of PANDAR regulatory network, mediates its dynamic control of malignant phenotype of GC cells.

However, our study demonstrates that the PANDAR/miR-637 axis in GC cell lines, the findings remain confined to in vitro systems. The absence of in vivo validation limits the translational relevance of these results. Patient-derived xenograft (PDX) models can more authentically simulate the growth patterns, pathological microenvironment, and drug responses of human tumors, significantly enhancing the clinical translational reliability of research conclusions. In previous studies, the PDX model was utilized to investigate the metastasis behavior of breast cancer [32] and the metabolic regulation of colorectal cancer [33], providing a condition similar to that of human tumor microenvironments for experiments. Additionally, PDX models can also be used to modulate miRNA function in vivo: Lee SJ et al. utilized PDX models to demonstrate that hsa-miR-CHA2 exerts anti-cancer effects by inhibiting Cyclin E1 [34], validating the feasibility of dissecting molecular mechanisms under physiologically relevant conditions. Therefore, we speculate that PDX models can be used to further explore the mechanism of action of PANDAR in GC, and plan to validate key findings using PDX models in future studies to enhance the clinical applicability of our conclusions. Although miR-637 was characterized as a downstream effector of PANDAR, the full spectrum of downstream effectors such as pathways regulated by miR-637, remains uncharacterized. Previous studies have demonstrated that miR-637 inhibited PTEN/AKT signaling pathway by targeting P-REX2a, and PTEN/AKT pathway plays a key role in the occurrence and development of GC [35]. So, we suspect that PTEN/AKT signaling pathway also be related to the regulation of PANDAR on GC. Additionally, studies have shown that miR-637 can also regulate the proliferation and invasion of GC cells by targeting other genes such as circ-NOTCH1 [36] and circRELL1 [20]. These findings reveal that miR-637 participates in gastric cancer progression through parallel pathways, providing a new perspective for precision therapy in gastric cancer. In the future, evaluating the activity of multi-target pathways based on miR-637 expression levels may offer richer biomarkers and therapeutic targets for the treatment of GC patients. In 2024, Ou L et al. shown that Helicobacter pylori infection as a major risk factor for GC [27]; however, whether this pathogen contributes to the development and progression of GC by regulating miR-637 expression remains unclear and warrants further investigation. In addition to its role in early diagnosis, our biomarker research based on PANDAR also holds significant potential in the discovery of new therapeutic targets for GC. By elucidating the molecular regulatory mechanism of PANDAR targeting miR-637 on the progression of GC, it provides information for the discovery of new therapeutic targets. Moreover, traditional medicine is also of great significance in oncology, playing an important role in enhancing therapeutic efficacy through complementary mechanisms, reducing side effects, or improving the overall condition of patients. Studies have shown that certain traditional Chinese medicine compounds exert anti-cancer effects through mechanisms such as ion channel regulation or DNA damage pathways [37, 38], and these mechanisms may be related to the miRNA regulatory network. And there are also studies that further prove that the combination of traditional Chinese medicine with biomarkers can improve the accuracy of treatment [39, 40]. Therefore, future research can also combine the PANDAR/miR-637 axis with certain Chinese herbal components to enhance the therapeutic effect through combined therapy.

## Conclusion

In summary, lncRNA PANDAR is overexpressed in GC, and promotes the development of GC as a cancer-promoting factor. lncRNA PANDAR can be used as a highly sensitive biomarker for diagnosis and risk prediction of GC. PANDAR modulates the proliferation, migration and invasion of GC cells by negatively regulating miR-637.

## Data Availability

The datasets used and/or analysed during the current study are available from the corresponding author on reasonable request.

## Abbreviations

AFP: Alpha-fetoprotein
GC: Gastric cancer
HBsAg: Hepatitis B surface antigen
LncRNAs: Long noncoding RNAs
miRNAs: microRNAs
NIH: National Institutes of Health
PDX: Patient-derived xenograft
VGSCs: Voltage-gated sodium channels
